# Phytogenic Compounds as Alternatives to In-Feed Antibiotics: Potentials and Challenges in Application

**DOI:** 10.3390/pathogens4010137

**Published:** 2015-03-23

**Authors:** Chengbo Yang, M .A. Kabir Chowdhury, Yongqing Hou, Joshua Gong

**Affiliations:** 1 Jefo Nutrition Inc., 5020 Jefo Avenue, C.P. 325, Saint-Hyacinthe, QC J2S 7B6, Canada; E-Mails: cyang@jefo.ca (C.Y.); kchowdhury@jefo.ca (M.A.K.C.); 2 Hubei Key Laboratory of Animal Nutrition and Feed Science, Wuhan Polytechnic University, Wuhan 430023, China; E-Mail: houyq@aliyun.com; 3 Guelph Food Research Centre, Agriculture and Agri-Food Canada, 93 Stone Road West, Guelph, ON N1G 5C9, Canada

**Keywords:** phytogenic compound, organic acid, antibiotics, gut chemosensing, anti-inflammation, quorum sensing

## Abstract

This article summarizes current experimental knowledge on the efficacy, possible mechanisms and feasibility in the application of phytogenic products as feed additives for food-producing animals. Phytogenic compounds comprise a wide range of plant-derived natural bioactive compounds and essential oils are a major group. Numerous studies have demonstrated that phytogenic compounds have a variety of functions, including antimicrobial/antiviral, antioxidative and anti-inflammation effects and improvement in the palatability of feed and gut development/health. However, the mechanisms underlying their functions are still largely unclear. In the past, there has been a lack of consistency in the results from both laboratory and field studies, largely due to the varied composition of products, dosages, purities and growing conditions of animals used. The minimal inhibitory concentration (MIC) of phytogenic compounds required for controlling enteric pathogens may not guarantee the best feed intake, balanced immunity of animals and cost-effectiveness in animal production. The lipophilic nature of photogenic compounds also presents a challenge in effective delivery to the animal gut and this can partially be resolved by microencapsulation and combination with other compounds (synergistic effect). Interestingly, the effects of photogenic compounds on anti-inflammation, gut chemosensing and possible disruption of bacterial quorum sensing could explain a certain number of studies with different animal species for the better production performance of animals that have received phytogenic feed additives. It is obvious that phytogenic compounds have good potential as an alternative to antibiotics in feed for food animal production and the combination of different phytogenic compounds appears to be an approach to improve the efficacy and safety of phytogenic compounds in the application. It is our expectation that the recent development of high-throughput and “omics” technologies can significantly advance the studies on the mechanisms underlying phytogenic compounds’ functions and, therefore, guide the effective use of the compounds.

## 1. Introduction

Young animals are highly susceptible to various stressors, including bacterial diseases, resulting compromised growth performance and high mortality [1]. Antibiotics have long been used at sub-therapeutical levels in swine, poultry, aquaculture and ruminant diets for better growth performance [2,3]. However, the use of antibiotics as growth promoters in animal feed is becoming limited due to increased public concerns over the spread of antibiotic resistance in zoonotic bacterial pathogens, which poses a threat to public health. There have been a significantly increased number of studies focused on searching alternatives to antibiotics with similar antimicrobial and growth-promoting effects without inducing bacterial resistance and potential side effects to animals. Organic acids [4,5], enzymes [6], probiotics [7], prebiotics [8], antimicrobial peptide [9] and phytogenic compounds [2,10,11,12] have been widely recognized as potential alternatives to antibiotics in feed. 

Phytogenic compounds are defined as plant-derived natural bioactive compounds with positive effects on animal growth and health, and are often applied to essential oils (EOs), botanicals and herbal extracts [13]. Some phytogenic compounds are known to have antimicrobial, antiviral, antifungal and antioxidative properties [14], and traditionally been used as complementary or alternative medicines to improve human health or cure human diseases [15]. With the identification of active components of phytogenic compounds and some progresses in the mechanistic studies of these components in animals, there have been increased research efforts to use phytogenic compounds to substitute antibiotics in animal diets [2,16]. However, the results from these studies have largely been inconsistent [17,18,19] and the mechanisms are still inconclusive with limited resources and understandings [20].

Besides the inconsistent results and inconclusive mechanisms, the challenges of using phytogenic compounds in animal feed may also include side effects, regulatory obstacles and cost-effectiveness. Nevertheless, application of novel technologies integrating research to simultaneously examine genomes, metagenomes, transriptomes and proteomes should help to better understand the modes of action of phytogenic compounds and subsequently lead to the development of feasible and cost-effective ways to use phytogenic compounds in animal feed.

## 2. Antimicrobial Effects of Phytogenic Compounds

Phytogenic compounds play an important role in mediating interactions between the plant and environment. Among the phytogenic compounds produced by plants as secondary metabolites, EOs are some of the most studied compounds characterized by their strong odors [21,22]. Other less studied molecules are: acids, alcohols, aldehydes, acylic esters, sulfur containing compounds, coumarins and homologues of polypropanoids.

The biosynthetic machinery of bacterial cell walls has been the most target site by antimicrobial compounds [23,24]. EOs and their derivatives can act against both cell walls (including membranes) and cytoplasm, often completely changing the morphology of the cells. Because of these properties, EOs are regarded as potential alternatives to antibiotics in animal production systems [25,26,27]. EOs increase the permeability of bacterial cell membranes resulting in cell contents leakage and eventually killing the cell. The leakage usually occurs through cell wall degradation, cytoplasmic membrane damage, cytoplasm coagulation and membrane proteins destruction [28,29,30,31] as well as the proton motive force reduction [32], where the EOs could play a decidedly active role.

### 2.1. Minimum Inhibitory Concentration (MIC) of Various Phytogenic Compounds

Minimum inhibitory concentration (MIC) can be described as the lowest concentration of an antimicrobial agent or chemical that inhibits the growth of a microorganism after incubation. A MIC is regarded as the most basic laboratory measurement of the activity of an antimicrobial agent against a microorganism. The MIC of phytogenic compounds is usually determined against a target bacterium by broth or agar macro-dilution method. Sometimes, sub-MICs are selected for the assessment of anti-quorum sensing (QS) activity of the test strains. The MIC of each phytogenic compound can vary from bacterium to bacterium and, in case of a single species, from strain to strain. The conditions for MIC assays can also influence the results. Table 1 (At the end of the main text, above the “Conclusions”) describes some commonly used phytogenic compounds and their MIC values on different bacterial species.

### 2.2. Synergy of Phytogenic Compounds and Organic Acids

Approximately 90–95% of the cell walls of Gram-positive (G^+^) bacteria are comprised of peptidoglycan allowing hydrophobic molecules to easily penetrate the cells and to act on both the cell walls and the cytoplasm. Upon entering the cell, phenolic compounds can interfere with enzymes involved in the production of energy at lower concentrations, and denature proteins at higher concentration. On the other hand, peptidoglycan layer in Gram-negative (G^−^) bacteria is only 2–3 mm thick and composes only 20% of the dry weight of the cell. An outer-membrane lies outside of this peptidoglycan layer, composed of a double layer of phospholipids firmly linked by Braun’s lipoprotein to the inner membrane. Generally, G^−^ bacteria are more resistant to EOs than the G^+^ bacteria. The presence of thick outer membrane in G^−^ bacteria makes them less permeable providing an extra layer of protection [33]. The core polysaccharides and the O-side chain, provides the “quid” allowing these bacteria to be more resistant to EOs and other natural extracts with anti-microbial properties.

Organic acids seem to be more effective against G^−^ bacteria than EOs [34,35,36]. In a study comparing the effects of grape seed extract (GE), citric acid (CA) and lactic acid (LA) on *Vibrio* *parahaemolyticus* in sucked oysters, Mahmoud (2014) reported a much higher MIC value of GE (10 mg/mL) compared to those of CA (5 mg/mL) and LA (1 mg/mL) [36]. Small hydrophilic solutes of organic acids are able to pass through the membrane via porin proteins but not the hydrophobic polyphenol molecules. The mechanism of inhibition to microorganisms by organic acids is related to several factors such as reduction in pH, the ratio of the un-disassociated form of the acid, chain length, degree of branching and cell physiology/ metabolism [37]. Weak organic acids are lipophilic in nature, can easily penetrate the plasma membrane and thus acidify the cell’s interior eventually killing the bacterium [38].

Several studies reported additive effects of some EOs and organic acids [35,39]. Zhou *et al.* (2007) reported an EO (carvacrol or thymol) in combination with some organic acids (acetic acid or citric acid) but not with the lactic acid worked better against G^−^ bacteria, *Salmonella typhimurium*, than individual EOs or organic acids [35]. A similar effect was observed with multiple strains of *Salmonella*, *Listeria monocytogenes*, *E. coli* and *Streptococcus aureaus* when treated with oregano oil in combination with caprylic acid [39]. The reason behind the additive effects of some EOs and organic acids is not well understood. However, it is known that phenols in EOs target bacterial cell membrane by changing its structure and function, resulting in swelling and thus increasing the membrane permeability [40]. The damage to the cell membrane might explain the observed additive effects, since the phenolic compounds could cause sublethal injury to cell membranes, increasing the susceptibility of the bacteria to acidic environment. Moreover, at low pH the hydrophobicity of an EO increases, enabling it to more easily dissolve in the lipids of the cell membrane of target bacteria [41]. In recent studies has been clearly shown *in vivo* efficacy of such complementary dietary strategies in broilers [42,43].

### 2.3. Mode of Action of Essential Oils

The actions of EOs on microbial cells vary by the location of their functional hydroxyl or alkyl groups. For example, thymol and carvacrol, the two common terpenoids, have similar antimicrobial effects but act differently against G^+^ or G^−^ bacteria based on the locations of one or more functional groups in these two molecules. The hydroxyl group of the phenolic terpenoids and the presence of delocalized electrons are important elements for their antimicrobial action [26,31]. The locations of the functional groups such as hydroxyl groups in these molecules usually determine the level of their activity on different bacteria. Hellander *et al.* (1998) reported that thymol and carvacrol had prominent outer membrane-disintegrating properties, due mainly to their enhanced ability to release lipopolysaccharides, which acted more like detergents for sensitization [30]. Because of the lipopolysaccharide release properties, both thymol and carvacrol have shown superior antimicrobial properties against some G^−^ bacteria than other phytogenic compounds.

## 3. Improvement of Feed Palatability, Feed Intake and Feed Digestibility

Phytogenic compounds have the potential to increase feed intake by improving the palatability of diet resulting from the enhanced flavor and odor, especially with the use of EOs [44]. However, the reported effect of supplemented EOs to pig diets on animal feed intake is highly variable [2,45,46,47]. Moreover, improving feed palatability is not applied to poultry because the birds are not sensitive to odor [48], although phytogenic compounds have been widely used in poultry diets with positive results in growth performance [14,49,50]. It was comprehensively reviewed in two recent reports that it could be easily seen highly variable feed intake responses to feed fortified with plant bioactives in broilers [51] and layer hens [52] which were similar to that observed in pigs. It has been suggested that the observed increase in feed palatability associated with the addition of EOs could be also due to their antioxidative effects, which might contribute to preserving the qualities of diets and preventing the release of unfavorable odors [53,54]. In this regard, it might be interesting to replace the antioxidants commonly used in the animal diet, such as ethoxyquin and butylated hydroxytoluene, with sufficient amounts of phytogenic compounds (natural antioxidants), especially when chemical antioxidants are prohibited. Interestingly, there is little or no evidence of better palatability in fish or shrimp fed diets containing phytogenic compounds such as carvacrol or thymol [55,56]. Despite no differences in feed intake compared to the control diets, Giannenas *et al.* [2012] reported significantly higher feed efficiencies (0.58 and 0.63) in rainbow trout fed diets containing carvacrol and thymol, respectively, compared to those fed the control diets (0.56) [55].

The gastrointestinal tract presents the largest and most vulnerable surface to outside world and it is not only the main site of nutrient absorption but also the main place of chemosensory system with many nerve and receptors [57]. Recently, there has been an increased interest in the gut chemosensory system due to the fact that the system can regulate digestion, absorption and metabolism and also has nutritional and pharmacological applications in improving gut development and health [58]. The gut epithelium has approximately 90% of the absorptive epithelial cells with the expression of nutrient transporters [59] and enteroendocrine cells for secreting gut peptides including glucoinsulinotropic polypeptide, glucagon-like peptides 1 and 2 and peptide YY [60]. Recent studies indicated that taste receptors, such as the sweet taste receptor T1R1+T1R3, the umami taste receptor T1R1+T1R3 and bitter taste-sensing type 2 receptors (T2Rs) are not only located in the taste buds but also evident in the gut [61,62]. Moreover, there are also the amino acid-sensitive calcium sensing receptor (CasR) and lipid receptors (GPR40, GPR119, and GPR120) in the gut [64]. All these receptors belong to a group of G protein coupled receptors [64]. The main functions of nutrient transporters are to absorb nutrients from the lumen of gut. Interestingly, a recent study indicates that nutrient transporters also contribute to the detection of nutrient in the lumen as transceptors [65]. Therefore, nutrients including amino acids, peptides, sugars and lipids can be detected by the chemosensors. One can also assume that other chemicals in feed can also be detected by the gut chemosensors. The chemosensors transduce information regarding the nutritional profile of the lumen to regulate nutrient transporter expression, digestive secretions and gut peptide secretion, ultimately to control feed intake, digestion, absorption and metabolism. It has been reported that phytogenic compounds can regulate the gene expression profile of ileal mucosa [19,20] and stimulate digestive secretions for improving nutrient digestibility [16]. However, the mechanisms on how to regulate gene expression relating to immune and digestive functions are still not fully understood. We may expect that the effect of phytogenic compounds can be mediated through a specific receptor in the gut, like the green tea polyphenol EGCG receptor [66]. Identification of specific receptors for phytochemical compounds would significantly improve our understanding of the underlying mechanisms.

## 4. Anti-Inflammation Effect of Photogenic Compounds

The gut displays various functions including absorption of nutrients, absorption and secretion of electrolytes and water, secretion of mucin and immunobulins and selective barrier protection against harmful antigens and pathogens [67]. In the past decade, the concept has emerged that, in addition to its absorption, secretion and barrier properties, the gut plays an active role in organ integrity and body defense [68,69]. Gut epithelial cells act as “watch dogs” for immune system. They can signal the onset of the host innate and acquired immune responses or inflammation through cytokines that are crucial for the recruitment and activation of neutrophils, macrophages, T and B cells and dendritic cells [68,69]. The gut immune system is quite different from systemic immunity, in that the gut mucosa immune system must balance two opposing functions: to mount an immune response to pathogens, while maintaining tolerance to antigens derived from commensal bacteria and food/feed [69]. The imbalance of those two opposite functions causes malfunction, such as food intolerance, inflammation and diseases. Gut inflammation has adverse effects on gut growth and the efficiency of nutrient utilization. Numerous studies have demonstrated that gut inflammation and chronic inflammatory diseases are associated with gut morphology changes, mucosa damage, enhanced mucosal permeability, poor gut development and reduced nutrient absorption capacity [70,71,72,73]. Basically, there are three types of gut inflammation, including infection-associated, diet allergy-associated and weaning-associated gut inflammation. Although the inflammation does not result in the full-blown clinical symptoms, it leads to a severe reduction of performance and causes economic loss.

In the early stages of immune response, macrophages enter the affected tissues and produce a strong inflammatory reaction, and then T cells are also involved in the promotion of inflammation in later stages. During the inflammatory process, the transcriptional factor nuclear factor kappa B (NF-kB) plays a very important role [74]. After activation by various inducers including cytokines, reactive oxygen species (ROS) and bacterial lipopolysaccharides, NF-kB translocates from the cytoplasma to the nucleus and induces the synthesis of a wide variety of pro-inflammatory proteins including cytokines, chemokines, adhesion molecules or enzymes involved in the inflammation, apoptosis and cell proliferation [75]. As a counterpart of NF-κB, nuclear factor-erythroid 2-related factor-2 (Nrf2)–a redox sensitive transcription factor, which under normal conditions is sequestered in the cytoplasm by Kelch-like ECH-associated protein 1 (Keap1), is dissociated from Keap1 and translocated into the nucleus, activating transcription of genes containing an antioxidant response element (ARE) [76]. The Nrf2-ARE pathway positively regulates the expression of antioxidant and detoxification enzymes, including superoxide dismutase (SOD), catalase (CAT), glutathione peroxidase (GPx), glutathione S-transferase (GST), glutathione reductase (GR), NADH(P)H-Quinone-Oxidoreductase 1 (NQO1), Heme oxygenase (HO1) and the glutathione (GSH) precursor gamma-glutamyl cysteine synthetase (γ-GCS), in an effort to re-establish cellular redox homeostasis [77,78,79,80]. There is increasing evidence of potential cross-talk between the Nrf2 and NF-κB pathways, with Nrf2 gene disruptions increasing susceptibility to inflammatory conditions [81]. Since gut inflammation in animals not only impairs function and integrity of the gut but also affects growth performance, dietary strategies to inhibit the inflammatory process in the gut are in great demand. Phytogenic compounds have been shown to manipulate the Nrf2 and NF-κB transcription factors to provide oxidative stress defense and suppress inflammation both *in vitro* [83,84]. A number of studies have demonstrated that phytochemicals including curcumin [85], caffeic acid phenethyl ester (CAPE) from honeybee propolis [86], epicatechin [87], a grape seed and grape marc extract (GSGME) [83], cinnamaldeyde [82] and anthocyanins from purple sweet potato [88] increased the expression or translocation of Nrf2 and reduced or inhibited the activation of NF-κB, suggesting that phytogenic compounds which modify the Nrf2 and NF-κB pathways can protect against oxidative stress and reduce inflammation, and eventually lead to the improvement of animal health and growth performance. Fiesel *et al.* (2014) reported that a down-regulation of several pro-inflammatory genes in the mucosa of various parts of the gut might contribute to the increased feed efficiency observed in the pig fed polyphenol-rich plant products [84]. A recent study indicated that dietary supplementation with cinnamon oil alleviated LPS-induced injury by suppressing inflammation [89]. On the other hand, three phytochemicals (carvacrol, cinnamaldehyde, and capsicum) at low doses presented immune-enhancing properties that could protect broiler chickens against live coccidiosis challenge infection [90]. A more recent study indicated that inclusion of alkaloid sanguinarine in weaner feed has beneficial effects on their growth performance and stimulates anti-inflammatory acitivity [91]. In view of these facts, it is clear that phytochemicals can modulate animal immune response through different pathways to enhance animal health.

## 5. Quorum Sensing (Bacterial Cell Signaling)

### 5.1. Bacterial Cell Signaling

One of the recent significant discoveries in microbiology is bacterial communication and its mechanism through cell-cell signaling (quorum sensing [QS]). The QS has a role in the regulation of a wide variety of different physiological processes, especially the virulence factors which are important during the process of pathogenic bacterium-host interactions [92,93,94,95]. Three major groups of signaling molecules have been identified for quorum sensing, acyl-homoserine lactones (AHL), small polypeptides, and autoinducer-2 (AI-2). AHLs are biosynthesized by members of the LuxI family of AHL synthases and mainly used by G^−^ bacteria [96]. G^+^ bacteria do not harbor LuxI or LuxR homologues and instead utilize modified oligopeptides as autoinducer molecules [97]. AI-2 can be shared by both G^−^ and G^+^ bacteria and the gene, *luxS*, is responsible for the synthesis of AI-2 in all bacteria that produce AI-2 [98]. The QS has been shown to play significant roles in the regulation of virulence factors in several enteric pathogens [94,99,100,101]. Therefore, disrupting QS by small molecules or QS quenching enzymes can be a promising tool to control enteric pathogens in food-producing animals. However, developments in the identification of QS inhibitors and studies on their mechanisms and application are still required.

### 5.2. Disrupting Quorum Sensing 

Although each quorum-sensing circuit used by a given bacterium is unique, all QS systems have a similar mechanism comprised of signal synthesis, signal accumulation and signal detection [102]. Therefore, QS inhibitors have been found to target at least one of these three steps: inhibition of QS signal biosynthesis, QS signal degradation and inactivation, and inhibition of signal detection [102,103]. There have been extensive studies to investigate the synthesis and use of small molecules to disrupt QS-regulated virulence gene expression. A number of natural and synthetic small molecule inhibitors of QS have been reported [104]. Halogenated furanones are amongst the most intensively studied QS-disrupting compounds [105]. In addition to the use of small molecules, an alternative strategy for inhibiting QS is enzymatic degradation of QS autoinducers [103]. There are three known classes of enzymes (lactonases, acylases and oxidoreductases), which can hydrolyze AHLs to produce products that are no longer active signaling agents [106]. A very recent study with gnotobiotic mice model indicated that *Ruminococcus obeum* could reduce *Vibrio* *cholerae* colonization through increased LuxS*/*AI-2-based QS of *Ruminococcus obeum* [107]. Taken together, synthesized or natural small molecules, QS quenching enzymes and specific probiotics bacteria could be used to limit the colonization of enteric pathogens through the mechanisms to disrupt QS and it is a promising approach to control diarrheal diseases.

### 5.3. Effect of Phytogenic Compounds on Quorum Sensing

There is evidence supporting QS disrupting ability of various phytogenic compounds, including extracts from alfalfa seeds [108,109,110], herbs [111,112,113,114] and essential oils [115,116,117]. However, all the evidence has been generated from *in vitro* studies and the studies relating to food. The use of inhibition of QS to control bacterial infections in food-producing animals is an unexplored strategy with the exception of aquaculture [118]. As the first case reported by Manefield *et al.* (1999) [119], furanone extracted from Australian microalge, *Delisea pulchra*, increased the survival of brine shrimp larvae that had been challenged with different pathogenic isolates of *V. campbellii*, *V. harveyi* and *V. parahaemolyticus* [105]. Further research indicated furanone can block HAI-1- and AI-2-mediated signalling in *V. harveyi* by decreasing the DNA-binding activity of the QS transcriptional regulator LuxR_Vh_ [120]. However, furanone is toxic to higher classes of organisms with a ratio of 2.5–4.0 between toxic and therapeutic concentrations [121,122]. Organic compounds such as cinnamaldehyde and its derivatives have been reported to be effective against *V. harveyi* in brine shrimp [123,124] and *Macrobrachium rosenbergii* larvae [125] as well as against *Aeromonus hydrophila* and *A. salmonicida* in burbot (*Lota lota* L.) larvae [126] with a proposed mechanism of disrupting of protein-DNA interactions of the QS-responsive master regulatory protein LuxR. Although data pertaining to the use of QS inhibition in animal pathogens, including the impact of QS on their virulence, are lacking, it is expected that this area will receive considerable attention in the coming years.

## 6. Challenges and Prospective of Phytogenic Compounds in Animal Feed

### 6.1. Challenges of Using Phytogenic Compounds in Animal Feed

A viable alternative to in-feed antibiotics is expected to be safe to the public, cost-effective in production, and friendly to the environment [12]. Because of these multiple requirements, no single alternative has been developed in the field thus far that can fully replace antibiotics in feed.

It is difficult to conduct systematic and comprehensive evaluations toward the efficacy and safety of phytogenic compounds due to their complex composition. In addition, challenges in using phytogenic compounds as animal feed additives may include potential side effects (toxic, unpleasant odor/taste), regulatory affair concerns and possible interactions with other feed ingredients [26,127,128]. There have hardly been developed analytical methods to identify and quantify the traceability of phytogenic compounds in feeds and animal tissues. Although most phytogenic compounds are a group of natural alternatives to antibiotics and are generally recognized as safe (GRAS) by the Food and Drug Administration of the United States [129], a complete assessment on the toxicity and safety of phytogenic compounds is still needed before the compounds can be used extensively in animal feeds.

Fully understanding the mechanism on how in-feed antibiotics can promote animal growth is important to the development of effective alternatives to antibiotics. There have been few hypotheses on the mechanisms, including the inhibition of pathogens (reducing infection), reduction of total bacterial burden in the gut (making more energy and nutrients available for animals), thinning of the gut mucosal layer (increasing nutrient adsorption) and modulation of the immune system (reducing inflammation and infection) [3,130]. Although the hypotheses are supported by some evidence, a full understanding of the mechanisms is still lacking, which has limited our effort in developing effective alternatives including phytogenic compounds.

The gastrointestinal ecosystem is well organized and very complicated. It is mainly composed of the epithelial cells, mucosal immune system and microbiota, which is mainly composed of commensal and beneficial bacteria as well as bacterial pathogens sometimes. The ecosystem is normally in homeostasis. Any change that disturbs the homeostasis would alter gut functions and thus undermine gut health and even animal growth and well-being. As described above, phytogenic compounds have multiple functions as whole, including antimicrobial and antioxidant activities as well as digestion- and immune-enhancing properties. To define the specific effect and target site (either animal host or its microbiota) of individual phytogenic compounds remains critical and will facilitate the application of phytogenic compounds in feed.

Phytogenic compounds have been investigated extensively as antibiotics alternatives. However, the results from previous studies are highly variable. There appear to be four reasons associated with the inconsistency: (i) variations in the composition of phytogenic compounds due to plant growing locations, manufacturing methods and the storage conditions [129,131]; (ii) variations in the dosages that may not be efficacious [132]; (iii) the lack of efficacy of phytogenic compounds; (iv) varied conditions during the trials such as environment, animal age, genetics, feeds and health status [133]. In addition, the MIC values of most phytogenic compounds are significantly higher than the levels to be acceptable in animal production from the viewpoint of cost-effectiveness, since the acceptance by the industry to use feed additives to optimize the animal performance and health is largely dependent on feed input costs.

Some phytogenic compounds are very volatile and can evaporate rapidly, leading to largely varied final concentrations in the products [26]. The stability of phytogenic compounds during feed processing is often questionable. In addition, several studies indicated that carvacrol, thymol, eugenol and trans-cinnamaldehyde were mainly or almost completely absorbed in the stomach and the proximal small intestine of piglets after oral injection [18]. Additionally, EOs may absorb to feed components, leading to a reduced antimicrobial activity [17]. Therefore, without proper protection, most EOs will be lost during feed processing and delivery to the animal gut and thus may not be able to reach the lower gut of animals where most pathogens reside and propagate. It will reduce the profitability of feed eventually and become one of major barriers for EO application in feed.

### 6.2. Prospective of Phytogenic Compounds in Animal Feed

A combined use of different antibiotic alternatives holds the most promising solution to replace antibiotics in feed. There are three major reasons: (i) an individual alternative is unlikely to cover all the performance-enhancing functions of antibiotics; (ii) there may be a synergistic effect between different alternatives to reduce required effective dosages, e.g., the synergistic effects between organic acids and essential oils; (iii) replacing antibiotics should be an integrated approach including biosecurity, nutrition and management rather than an supplementation of feed additives alone. More recently published studies have been shown that a combined use of different antibiotic alternatives had better effects on the health and performance of weaned pigs [134,135]. A better understanding of the effects and mechanisms of action of various alternatives will guide the designs for more effective phytogenic additives to improve feed efficiency and promote animal growth.

It is necessary to develop an effective and practically feasible delivery method for the use of EOs. Microencapsulation has become one of the most popular methods to solve the issue [136]. A commonly used method is the microencapsulation of EOs in a lipid matrix that could dissolve as it passes along the small intestine [136]. A recent study indicated that alginate-whey protein microparticles could be used as a target delivery carrier in feed to enhance the gut delivery of carvacrol in broiler chickens [137]. Furthermore, the effect of encapsulated carvacrol on reducing necrotic enteritis in *Clostridium perfringens*-challenged broilers was comparable to that of antibiotics in feed (unpublished data). Thus, it is clear that proper microencapsulation technologies can lower the required effective dosage of a phytogenic compound in feed and reduce the feed cost.

**Table 1:** Minimum inhibition concentration (MIC) of phytogenic compounds against various species of bacteria.

Product	Plant Species	Pathogenic microbe	Gram	MIC (unit)	MIC (#)	Reference
Thymol	Thyme	*Lactococcus piscicum*	+	mg/L	320	[22]
		*Streptococcus phocae*	+	mg/L	640	[22]
		*Flavobacteriaum psychrophilum*	-	mg/L	320	[22]
		*Vibrio anguillarum*	-	mg/L	80	[22]
		*V. parahaemolyticus*	-	mg/L	320	[22]
		*Pseudomonus sp.*	-	mg/L	640	[22]
		*Lactococcus lactis*	+	mg/L	1280	[22]
Proanthcyanids	Grape seed extract	*V. parahaemolyticus*	-	mg/mL	10	[36]
Eugenol	Clove	*Vibrio sp.*	-	ug/mL	156	[138]
		*Escherichia coli*	-	ug/mL	625	[138]
		*Salmonella*	-	ug/mL	156	[138]
		*Pseudomonas sp.*	-	ug/mL	325	[138]
		*Edwardsiella tarda*	-	ug/mL	56–125	[138]
		*Aeromonas hydrophilla*	-	ug/mL	625	[138]
Essential oil extract	Clove	*Vibrio sp.*	-	ug/mL	15	[138]
		*Escherichia coli*	-	ug/mL	31	[138]
		*Salmonella sp.*	-	ug/mL	62	[138]
		*Pseudomonas sp.*	-	ug/mL	62	[138]
		*Edwardsiella tarda*	-	ug/mL	31–62	[138]
		*Aeromonas hydrophilla*	-	ug/mL	15	[138]
Carvacrol	Oregano	*Listonella anguillarum*	-	ug/mL	25	[56]
Essential oil extract	Oregano	*Salmonella enteritidis*	-	uL/mL	50	[39]
		*E. coli*	-	uL/mL	51	[39]
		*Staphylococcus aureus*	-	uL/mL	48	[39]
		*Listeria monocytogenes*	-	uL/mL	52	[39]
Essential oil extract	Oregano	*Staphylococcus aureus*	-	uL/mL	125	[34]
Essential oil extract	Basil	*S. aureus*	-	mg/mL	2.5	[139]
		*L. monocytogenes*	-	mg/mL	2.5	[139]
		*Bacillus cereus*	-	mg/mL	1.25	[139]
		*E. coli*	-	mg/mL	1.25	[139]
		*S. typhimurium*	-	mg/mL	2.5	[139]
	Cinnamon	*S. aureus*	-	mg/mL	5	[139]
		*L. monocytogenes*	-	mg/mL	2.5	[139]
		*Bacillus cereus*	-	mg/mL	2.5	[139]
		*E. coli*	-	mg/mL	5	[139]
		*S. typhimurium*	-	mg/mL	5	[139]
	Oregano	*S. aureus*	-	mg/mL	5	[139]
		*L. monocytogenes*	-	mg/mL	2.5	[139]
		*Bacillus cereus*	-	mg/mL	2.5	[139]
		*E. coli*	-	mg/mL	5	[139]
		*S. typhimurium*	-	mg/mL	5	[139]
	Rosemary	*S. aureus*	-	mg/mL	10	[139]
		*L. monocytogenes*	-	mg/mL	2.5	[139]
		*Bacillus cereus*	-	mg/mL	5	[139]
		*E. coli*	-	mg/mL	2.5	[139]
		*S. typhimurium*	-	mg/mL	10	[139]

## 7. Conclusions

The practice without using antibiotic growth promoters in food animal production has been implemented in the European Union countries since 2006 and more countries are expected to follow. There are a number of challenges after the withdrawal of antibiotics from feed. The cost-effectiveness in substituting antibiotics with alternatives is the most challenging one, which remains critical for ensuring the long-term sustainable animal production. Phytogenic compounds have a large variety of active ingredients and thus represent one of the most promising alternatives to antibiotics. However, their application in food animal production has been limited, largely owing to their inconsistent efficacy and lack of full understanding the modes of action. A better understanding the effects of phytogenic compounds on the three components, gut microbiota, gut physiology and immunology, within the gastrointestinal ecosystem and the mechanisms behind will possibly allow us to make the best use of phytogenic substances for economically effective and sustainable animal production. Finally, the potential risks in the use of phytogenic compounds for animal production and to human health need to be evaluated even though phytogenic compounds are GRAS.
